# The Lung Microbiome in Young Children with Cystic Fibrosis: A Prospective Cohort Study

**DOI:** 10.3390/microorganisms9030492

**Published:** 2021-02-26

**Authors:** Barry Linnane, Aaron M. Walsh, Calum J. Walsh, Fiona Crispie, Orla O’Sullivan, Paul D. Cotter, Michael McDermott, Julie Renwick, Paul McNally

**Affiliations:** 1 Centre for Interventions in Infection, Inflammation and Immunity (4i) and Graduate Entry Medical School, University of Limerick, Limerick V94 T9PX, Ireland; barry.linnane@hse.ie; 2 National Children’s Research Centre, Our Lady’s Children’s Hospital, Crumlin, Dublin D12 N512, Ireland; paulmcnally@rcsi.ie; 3 Teagasc Food Research Centre, Moorepark, Fermoy, Co Cork P61 C996, Ireland; aaronbreathnach@hotmail.com (A.M.W.); Calum.Walsh@teagasc.ie (C.J.W.); fiona.crispie@teagasc.ie (F.C.); orla.osullivan@teagasc.ie (O.O.); Paul.Cotter@teagasc.ie (P.D.C.); 4 APC Microbiome Ireland, University College Cork, Cork T12 YN60, Ireland; 5 Pathology Department, Our Lady’s Children’s Hospital, Crumlin, Dublin D12 N512, Ireland; michael.mcdermott@olchc.ie; 6 Department of Clinical Microbiology, Trinity College Dublin, Trinity Centre for Health Science, Tallaght University Hospital, Dublin 24, Ireland; 7 Department of Paediatrics, Royal College of Surgeons in Ireland, Our Lady’s Children’s Hospital Crumlin, Dublin D12 N512, Ireland

**Keywords:** infection, inflammation, microbiota, microbiome, lung, cystic fibrosis, children, paediatrics, bronchoscopy, bronchoalveolar lavage

## Abstract

The cystic fibrosis (CF) lung harbours a diverse microbiome and reduced diversity in the CF lung has been associated with advancing age, increased inflammation and poorer lung function. Data suggest that the window for intervention is early in CF, yet there is a paucity of studies on the lung microbiome in children with CF. The objective of this study was to thoroughly characterise the lower airway microbiome in pre-school children with CF. Bronchoalveolar lavage (BAL) samples were collected annually from children attending the three clinical centres. Clinical and demographic data were collated on all subjects alongside BAL inflammatory markers. 16S rRNA gene sequencing was performed on the Illumina MiSeq platform. Bioinformatics and data analysis were performed using Qiime and R project software. Data on 292 sequenced BALs from 101 children with CF and 51 without CF show the CF lung microbiome, while broadly similar to that in non-CF children, is distinct. Alpha diversity between the two cohorts was indistinguishable at this early age. The CF diagnosis explained only 1.1% of the variation between the cohort microbiomes. However, several key genera were significantly differentially abundant between the groups. While the non-CF lung microbiome diversity increased with age, diversity reduced in CF with age. *Pseudomonas* and *Staphylococcus* were more abundant with age, while genera such as *Streptococcus, Porphyromonas* and *Veillonella* were less abundant with age. There was a negative correlation between alpha diversity and interleukin-8 and neutrophil elastase in the CF population. Neither current flucloxacillin or azithromycin prophylaxis, nor previous oral or IV antibiotic exposure, was correlated with microbiome diversity. Consecutive annual BAL samples over 5 years from a subgroup of children demonstrated diverse patterns of development in the first years of life.

## 1. Introduction

Cystic fibrosis (CF) is an autosomal recessive condition with impaired mucociliary clearance and innate airway defences [1]. Chronic airway infection and inflammation culminating in bronchiectasis are the main drivers of the morbidity and mortality of CF [1]. There is good evidence that these processes start early in life, with asymptomatic infants and pre-school children demonstrating clear associations between the presence of lower airway infection and inflammation and impaired lung function and structural airway changes [1,2].

Traditionally, CF airway infections have been known to be caused by a few well-known bacteria—the ‘usual suspects’. *Pseudomonas aeruginosa, Staphylococcus aureus, Haemophilus influenzae, Streptococcus pneumoniae* and *Stenotrophomonas maltophilia* are commonly cultured from CF airway specimens [3]. With the use of 16S ribosomal RNA sequencing, we now know that the CF airways are inhabited by a much more diverse microbial community including ‘emerging species’ and anaerobes never before associated with CF airway disease [4,5,6,7,8]. Perturbations in the CF airway microbiome are well described. However, it is unclear what effect these have on the “infection–inflammation–structural damage” model of CF lung disease [4,5,6,7,8,9,10,11,12,13]. Limited studies in young children with CF suggest that microbial diversity is initially low but increases with age [4,8,10,11,12,13]. In contrast, in older children and adults, diversity decreases with age and declining lung function [4,8,14,15]. In adults, with advancing lung disease and repeated exposure to antibiotics over an extended period, dominant CF pathogens emerge in a relatively fixed microbiome with limited diversity [5,7,16,17].

We aimed to describe the lower airway microbiome in clinically stable pre-school-aged children with CF, and, using prospectively collected clinical and biological data, explore relationships between microbiome composition and age, inflammation and antibiotic use.

## 2. Materials and Methods

This is a prospective observational cohort study, with cross-sectional and longitudinal components to sample acquisition and data analysis, and is reported in accordance with the STROBE statement [18]. 

### 2.1. Sample Collection

Bronchoalveolar lavage (BAL) was collected from infants and pre-school children undergoing routine BAL surveillance at three specialist CF centres in Ireland between 2010 and 2016 through the Study of Host Immunity and Early Lung Disease in CF (SHIELD CF), a prospective longitudinal study established around a clinical BAL surveillance programme. Specimens were collected from clinically stable children aged one to six years. Bronchoscopy was performed through a laryngeal mask airway (LMA) with no suction applied until below the carina. BAL was performed by instilling 1 mL/kg sterile 0.9% NaCl per aliquot (up to a maximum of 20 mL) and retrieved using low-pressure suction. The BAL was performed twice in the right middle lobe and twice in the lingula. All four samples were pooled. Clinical information regarding the status of the child on the day of the BAL and on their progress in the preceding year was recorded. Control subjects without CF undergoing bronchoscopy for clinical reasons, such as a previous haemoptysis, stridor or cough, were also recruited. No subjects were immunocompromised. These children were clinically well, as determined by the attending physician, at the time of the procedure. Negative sampling controls were performed on all bronchoscopes used in this study by washing 5 mL 0.9% NaCl through the decontaminated bronchoscopes prior to patient sampling. 

### 2.2. Processing of Clinical Samples

A standardised procedure was used across all three sites for sample acquisition, processing and storage as previously described [19]. Briefly, BAL was immediately sealed in a sterile container and transported to the laboratory on ice. An aliquot of BAL fluid was sent directly for standard microbiological culture. An aliquot of 1.5 mL of each BAL and the 5 mL negative sampling controls were mixed 1:1 with RNALater (Qiagen, Hilden, Germany) and frozen at −80 °C. The remaining amounts of samples were processed as follows: total cell counts were performed using trypan blue exclusion and differential cell counts were carried out manually on cytospin preparations of BAL, stained with haematoxylin and eosin to facilitate differential cell count by light microscopy. Interleukin-8 (IL-8) levels were determined by ELISA and neutrophil elastase (NE) levels were determined using an in-house activity assay. BAL was subsequently centrifuged at 1057× *g* and the supernatant aliquoted and frozen at −80 °C on site before cold-chain transfer to a central −80 °C biobank for long-term storage.

### 2.3. DNA Extraction and 16S RNA Gene Sequencing

The DNA extraction process was performed as previously described (Appendix A supplemental methods) [19] on all samples and negative sampling controls. DNA was normalised and 16S metagenomic libraries were prepared using primers to amplify the V3–V4 region of the 16S gene [20], with Illumina adaptors incorporate as described in the Illumina 16S Metagenomic Library Preparation guide. Molecular-grade water was used as template for negative procedural controls for the entire library preparation. The pooled libraries were assessed by an Agilent high-sensitivity DNA kit and quantified by qPCR using the Kapa Quantification kit for Illumina (San Diego, CA, USA). Libraries were then diluted, denaturated and sequenced on the Illumina MiSeq (Appendix A supplemental methods). Three patient samples were run on each sequencing run in order to determine the coefficient of variation between runs. 

### 2.4. Bioinformatics

Bioinformatic analysis was performed as previously described by Walsh et al. [21]. Briefly, Raw 16S rRNA gene sequencing reads were quality filtered using PRINSEQ with a sequence read length cutoff of 250 bp, min overlap of 20 bp and Phred score of q20. Denoising, operational taxonomic unit (OTU) clustering (97% identity), and chimera removal were performed using USearch (v7-64 bit). OTUs were aligned using PyNAST [22]. Alpha diversity and beta diversity were calculated using Qiime (1.8.0). Taxonomy was assigned using a BLAST search against the SILVA SSU 123 database [23,24]. Ribosomal Database Project (RDP) was also used to classify OTUs against GreenGenes database to corroborate findings using this method [25,26]. The R package decontam was run to assess contaminant OTUs. Replicates were assessed using Qiime unweighted Unifrac distance and PCoA models. Further bioinformatics was performed with the phyloseq package in R.3.2.2 [27].

Sequencing data have been deposited to the European Nucleotide Archive under the project accession number PRJEB28588.

### 2.5. Statistical Analysis

All statistical analysis was performed in R 3.2.2. The Kruskal–Wallis test was used to test for significant changes in alpha diversity between independent groups, while the Wilcoxon test was used to test for significant changes in alpha diversity within groups over time. The reported p-values were corrected for multiple comparisons using the Benjamini–Hochberg (BH) method. The Adonis function in the vegan package was used to perform permutational analysis of variance (PERMANOVA) to statistically assess the unweighted UniFrac dissimilarity between groups [28]. The Betadisper package was used to determine homogeneity in dispersion of variance between cohorts. Correlation analysis was performed using the Hmisc package [29]. Linear discriminant analysis (LDA) effect size (LEfSe) was used to identify differentially abundant taxa; an alpha value of ≤0.05 and LEfSe of ≥4.0 were used as the threshold for significance (Table S1) [30]. All data visualisation was performed using the pheatmap and ggplot2 package [31].

### 2.6. Ethics

Ethical approval was granted by the research ethics committee at Children’s Health Ireland (CHI) at Crumlin (GEN/210/11) and informed consent was obtained from the parents or guardians of all participating subjects.

### 2.7. Role of the Funding Source

The funding bodies had no direct role in this study. The corresponding author had full access to all the data and has final responsibility for the decision to submit for publication.

## 3. Results

### 3.1. Patient Characteristics and Sequencing Quality Control

A total of 336 BAL samples were taken from the biobank and, following quality control, 292 (87%) samples provided 16S rRNA gene sequencing reads of sufficient quality to proceed to analysis (Figure S1). These represented 152 patients; 51 controls (mean age 6.1 years), plus 101 subjects with CF (mean age 3.6 years) from whom there were 241 BALs. Within the CF cohort, 38 had one BAL, 22 had two BALs, 14 had three, 19 had four, seven had five and one had six (Table S3). Within the control cohort, all had one BAL. Baseline characteristics of the groups can be found in Table 1. The mean age of the CF cohort was lower, reflective of more children with CF having BAL samples taken when younger, with one centre performing routine BAL from one year of age. 

Sequencing generated an average of 198029 (32,493 to 201147) raw reads and an average of 178840 (29575 to 199709) high-quality reads across all samples after quality control. This corresponded to a total of 994 OTUs with an average of 90 (28 to 413) OTUs per sample. In order to establish whether any contamination was evident in our samples, we sequenced negative controls (Supplementary File F1) and the R package decontam, ran using the ‘combined’ method employing both prevalence in negative controls and frequency as a function of input DNA concentration, to identify contaminant OTUs (Supplementary File F2). Negligible contamination was identified. In true samples, contaminant OTUs represented a mean relative abundance of 0.8% and the two contaminant OTUs assigned to the genus *Pseudomonas* were responsible for a miniscule mean relative abundance of 0.07%. The coefficient of variation between runs based on the three samples repeated on each run was less than 1 (0.378 ± 0.0933). To further explore the consistency across our runs, the three replicate samples were analysed using unweighted Unifrac distance and PCoA models (one-way ANOVA, F statistic = 3.739, *p* = 0.06577). This satisfied the assumptions for PERMANOVA (R^2^ = 0.97203, *p* = 0.001). Therefore, 97.203% of the between-sample variance in this model is explained by the original sample. This shows great consistency of replicates over the runs. 

**Table 1:** Subject characteristics.

	CF	Control
Total	101	51
Female (%)	47(46%)	16(31%)
Mean age in years at time of bronchoscopy (±SD)	3.6 (± 1.8)	6.1 (±4.3) (*p* < 0.001)
Mean (±SD) total BAL cell count	13.8 (± 25.9) × 10^6^/L	6.3 (±16.4) × 10^6^/L (*p* = 0.02)
Mean (±SD) absolute neutrophil count	4.8 (± 11.0) × 10^6^ /L	1.3 (± 3.3) × 10^6^/L (*p* = 0.001)
Mean (±SD) IL-8 (pg/mL)	507 (± 297)	249 (± 243) (*p* < 0.001)
Mean Ln NE (ng/mL)	4.1 (± 1.5)	4.2 (± 0.9) (*p* = 0.55)
Genotype		Not applicable
F508del homozygous	58 (57%)	
F508del heterozygous	22 (22%)	
Other	9 (9%)	
No data	12(12%)	
BAL culture results (total #)	(*n* = 273)	(*n* = 51)
*P. aeruginosa* (% of total)	12 (4.4%)	1 (1.9%)
*S. aureus* (% of total)	55 (20.1%)	3 (5.8%)
*H. influenzae* (% of total)	67 (24.5%)	14 (27.4%)
*S. pneumoniae* (% of total)	24 (8.8%)	8 (15.7%)
*M. catarrhalis* (% of total)	8 (2.9%)	4 (7.8%)
*S. maltophilia* (% of total)	11 (4.0%)	1 (1.9%)
Pancreatic insufficient	81 of 83 with data 98%	Not applicable
Mean (±SD) weight z-score at time of bronchoscopy	−0.01 (±1.6)	No data
Mean (±SD) height z-score at time of bronchoscopy	−0.16 (± 2.1)	No data

Definition of abbreviations: SD = standard deviation, BAL = bronchoalveolar lavage, Ln = natural log, and NE = neutrophil elastase. # number.

### 3.2. Baseline Characteristics of the CF and Non-CF Lung Microbiome

**Figure 1:**
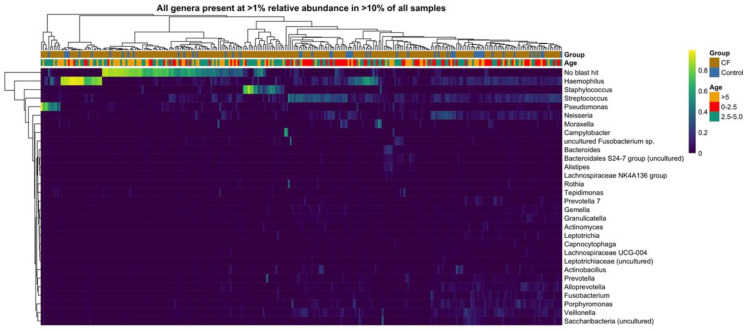
Heatmap of all genera present at ≥1% relative abundance in ≥10% of all samples.

Twenty-nine genera were detected at >1% relative abundance in >10% of samples in this study (Figure 1). This microbiome was dominated by well-known CF genera such as *Haemophilus, Staphylococcus, Streptococcus, Pseudomonas*, and regularly cultured genera, *Neisseria, Moraxella, Actinomyces, Porphyromonas*, and also emerging CF microbiome genera, *Fusobacterium, Bacteroides, Alistipes, Lachnospiraceae NK4A136 group, Rothia, Prevotella, Gemella, Granulicatella, Leptotrichia, Capnocytophaga, Lachnospiraceae UCG-004, Alloprevotella, Veillonella* and less regularly reported *Bacteroidales S24-7 group, Tepidimonas* and *Saccharibacteria*. A significant proportion, 21% of reads in the CF cohort and 15% in the control cohort, remained unassigned, having no match identified in the reference database (“unassigned”). In addition, at the genus level, multiple low abundance (<1%) genera cumulatively represent a significant proportion (26–27%) of the overall microbiome.

Overall, samples that were culture positive for important CF bacterial genera had higher relative abundance of those genera when sequenced. *Pseudomonas, Staphylococcus, Haemophilus, Achromobacter, Prevotella* and *Stenotrophomonas* genera were detected in higher mean relative abundance in samples that were positive for these genera by culture. *Veillonella* and *Streptococcus* were present in similar relative abundance across culture positive and culture negative samples (Supplementary Figure S2). For all genera, bar *Achromobacter* and *Stenotrophomonas*, sequencing detected genera in culture negative samples.

### 3.3. The overall Diversity of the CF and Non-CF Lower Airway Microbiome Is Broadly Similar with Significant Differences in Individual Species Abundance

**Figure 2:**
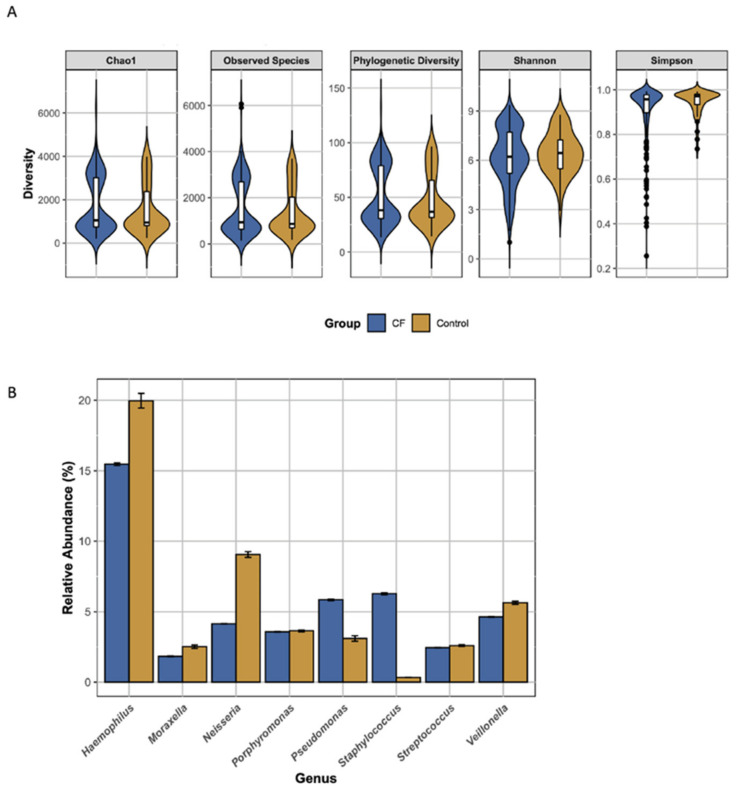
Comparison of the CF and control lung microbiome. (**A**) Violin plots of alpha diversity measures. (**B**) The percentage relative abundance of genera which were significantly differentially abundant between cohorts (i.e., LDA > 4) as determined by LEfSe (error bars show standard error).

There was no difference between the CF and non-CF microbiome in terms of overall alpha diversity when looking at Chao1, Simpson, Shannon, phylogenetic diversity and observed species (Figure 2A). In terms of beta diversity, unweighted Unifrac principle coordinate analysis (PCoA) and Betadisper analysis revealed that the dispersion of variance in the CF group was not heterogenous therefore limiting the assumptions that can be drawn from the PERMANOVA analysis. Overall the early CF and non-CF lower airway microbiome had similar alpha and beta diversity. To explore in more detail the differences between the cohorts, we identified taxa present at >1% relative abundance that were significantly altered between the cohorts (Figure 2B). Six of nine genera are more abundant in the control cohort, with *Haemophilus* and *Neisseria* the most prominent. The two exceptions are *Staphylococcus* and *Pseudomonas* which are present in significantly greater relative abundance in the CF lung.

These results suggest that infants and young children with CF, that are asymptomatic at the time of sample acquisition, have altered abundance of specific genera to that of similarly aged children without CF. Some of these genera are only emerging as common colonisers of the CF airway and their importance in CF warrants further investigation.

### 3.4. The CF and Control Airway Microbiomes Are Disparately Associated with Age in the First Years of Life

**Figure 3:**
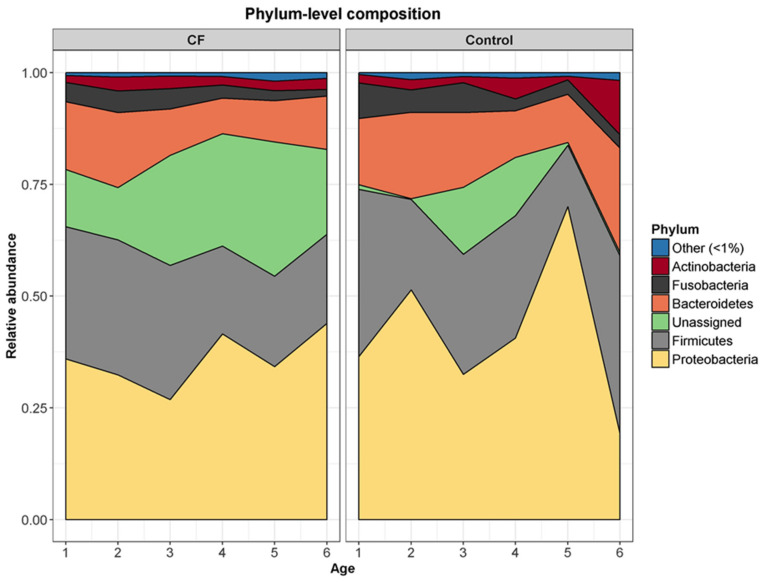
Cross sectional analysis demonstrating differences, with age, in the average phylum-level composition of the lower airway microbiota in CF and control BAL from 1 to 6 years of age.

We performed an age-matched cross-sectional comparison of phylum-level composition between the two cohorts (Figure 3 and Figure S3). Whilst the lung microbiome of both populations fluctuated with age, distinguishable patterns were evident in the cohorts. In the CF cohort, in the older children, there were overall increases in Proteobacteria (+8%) and unassigned phyla (+8%), while there were decreases in Bacteroidetes (−3%), Firmicutes (−10%), and Fusobacteria (−5%). In contrast, in the control cohort, there were overall increases in Actinobacteria (+10%), Bacteroidetes (+9%), and Firmicutes (+2%), while there were decreases in Fusobacteria (−5%) and Proteobacteria (−17%).

**Figure 4:**
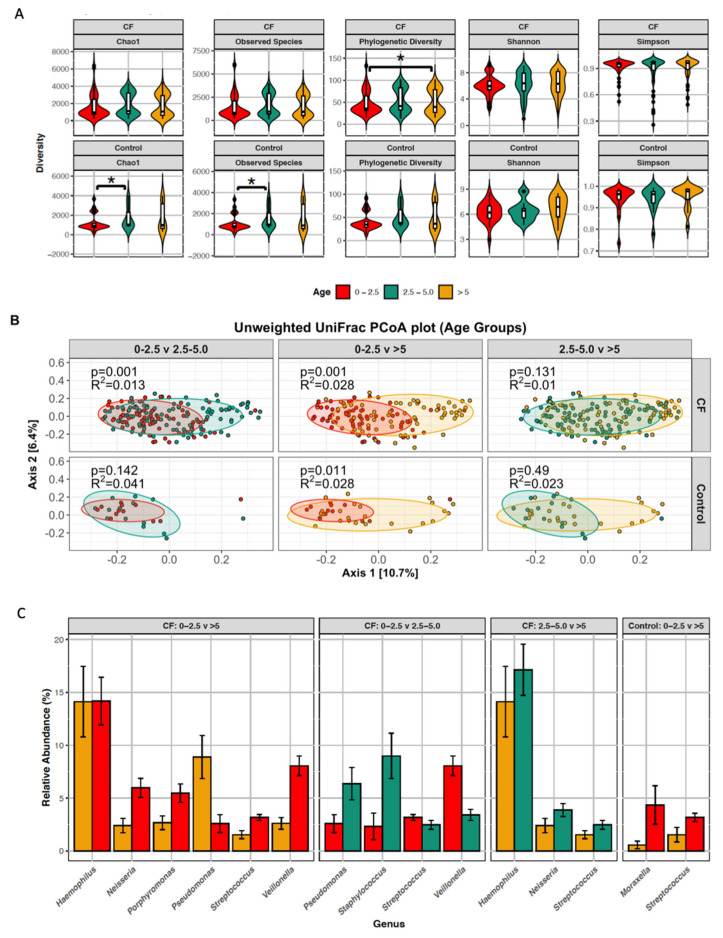
Age-associated changes in the microbiome of children with CF and without CF (control). (**A**) alpha diversity displayed as violin plots and (**B**) beta diversity displayed as unweighted UniFrac PCoA plots (ellipses illustrate 80% confidence intervals) of the lower airway microbiota across three age groups: 0 to 2.5 years old, 2.5 to 5 years old and over 5 years old. (**C**) Genera which were significantly differentially abundant (i.e., LDA >4.0) across these same groups, as determined using LEfSe.

To further explore the changes in the lung microbiome during early childhood, both cohorts were divided into three age groups: 0–2.5 years; >2.5–5.0 years; and >5 years (Figure 4). A significant decrease in diversity (PD whole tree) was observed with increased age in the CF cohort (*p* < 0.001) (Figure 4A). Conversely, a significant increase in diversity was observed with increased age in the control cohort (Chao1 *p* = 0.027 and observed species *p* = 0.043). With respect to beta diversity, there were significant dissimilarities in the CF microbiome between 0–2.5 year olds and 2.5–5 year olds (*p* = 0.001, *R*^2^ = 0.013) and again between the 0–2.5 year olds and >5 year olds (*p* = 0.001, *R*^2^ = 0.028) (Figure 4B). In the control cohort, there was significant dissimilarity between 0–2.5 year olds and >5 year olds only (*p* = 0.011, *R*^2^ = 0.028). Betadisper analysis revealed that there was no statistical difference between the homogeneity of variance of the age groups in the CF cohort and the control cohort (*F* = 1.3622, *p* = 0.2581 and F = 1.1836, *p* = 0.315, respectively).

The relative abundances of genera that are altered significantly between the age groups are presented in Figure 4C. In the control cohort, *Streptococcus* and *Moraxella* are significantly less abundant in the older children. This is in marked contrast to the CF lung where there are significant changes in the relative abundances of seven genera with age. *Pseudomonas, Staphylococcus* and *Haemophilus* are significantly more abundant in older CF children while *Streptococcus, Veillonella, Neisseria,* and *Porphyromonas* are significantly less abundant in older CF patients.

Taken together, our results suggest that, in the early years of life, the lung microbiome of children with CF is developing differently to that of controls.

### 3.5. The Longitudinal Development of the Lung Microbiome of Children with CF Is Highly Variable Within and Between Patients

**Figure 5:**
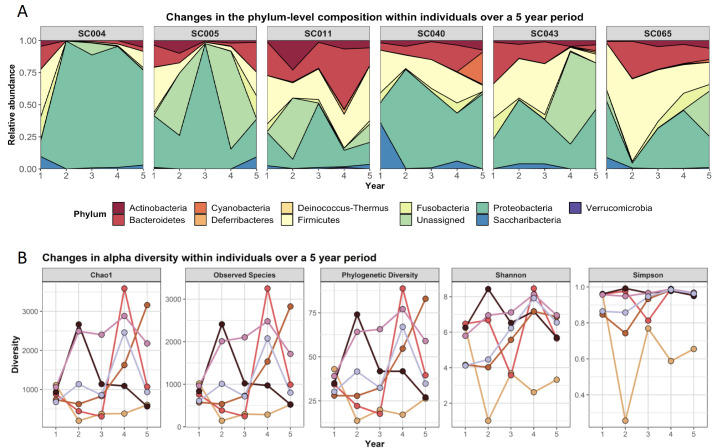
Longitudinal development of the CF airway microbiome over 5 years in six patients. (**A**) Relative phyla abundance and (**B**) alpha diversity in the CF lung microbiome.

Seven children with CF had a complete dataset, with BAL samples taken for five consecutive years from 1 to 5 years of age. This unique subgroup provides potentially important insights into the development of the lung microbiome in individuals with CF. Across all seven subjects the composition of the lower airway microbiome, even at phylum level, is highly variable year to year (Figure 5A), with no clear pattern being evident across the subjects. Indeed, even within subjects the composition and relative abundance alters markedly in each consecutive year (Figure 5A). In addition, Chao1, Shannon, and observed species measures of alpha diversity appear to increase over the first four years of life before decreasing in the fifth year. However, with the wide variability between patients and the low number of longitudinal samples in this study, this should be interpreted with caution (Figure 5B).

### 3.6. The CF Lung Microbiome Is Associated with Inflammatory Markers

Correlation analysis was performed to characterise the relationships between alpha diversity and inflammatory markers in the CF BAL samples (Table 2). Significant negative correlations were found between IL-8 and the Simpson (*R* = −0.21, *p* = 0.007) measures of alpha diversity. Similarly, significant negative correlations were found between NE and both the Simpson (*R* = −0.16, *p* = 0.037) diversity measures. No other significant correlations were identified. Our results indicate a potential link between decreased alpha diversity and increased inflammation in the CF lung.

### 3.7. Greater Antibiotic Use Was Not Associated with Altered Diversity

**Figure 6:**
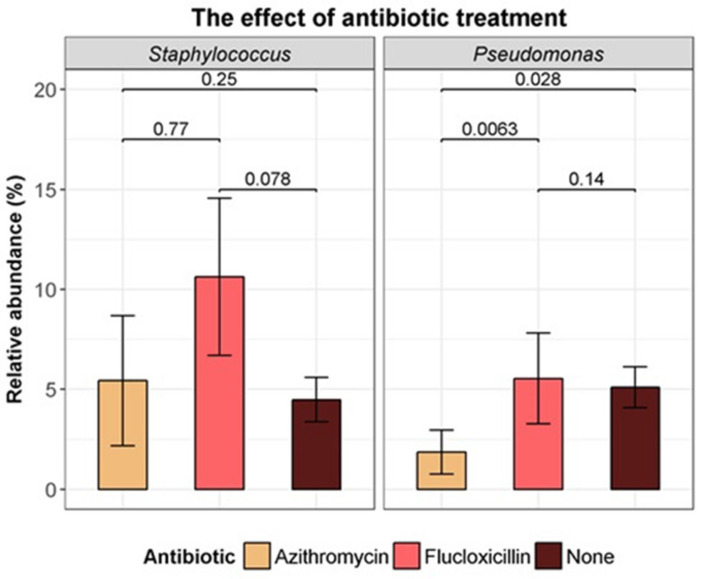
Comparison of relative abundance of Staphylococcus genus and Pseudomonas genus with flucloxacillin prophylaxis, and long-term azithromycin treatment, on the day of the bronchoscopy (Kruskal–Wallis test).

On the day of bronchoscopy, 33 (13.7%) patients were on prophylactic flucloxacillin and 21 (8.7%) were on long-term azithromycin treatment. There were no significant differences in alpha diversity measures between those treated with either flucloxacillin or azithromycin relative to those not on antibiotics on the day of the bronchoscopy (Table S2). While flucloxacillin use did not appear to significantly affect the relative abundance of *Staphylococcus* (*p* = 0.08) or *Pseudomonas* (*p* = 0.14), azithromycin use was associated with a lower relative abundance of *Pseudomonas* (*p* = 0.03) (Figure 6).

Regarding prior antibiotic exposure, the number of courses of oral antibiotics taken in the previous year ranged from zero to 12, with a median of four courses, resulting in a mean duration on oral antibiotics of 42 days (range 0 to 168 days). The number of admissions for IV antibiotics over the study period ranged from 0 to 6, with a median of 0 courses, representing a median of 0 days on IV antibiotics (range 0 to 43 days). Prior exposure to antibiotics did not appear to affect the lower airway microbiome as assessed by BAL during a time of clinical stability. No significant correlations were identified between total cumulative days on any (intravenous or oral) antibiotics in the previous year and alpha diversity measures (Table S2).

## 4. Discussion

This study presents the largest, robust description of the lower airway microbiome of young children with CF published to date, and describes changes in the microbiome with age. Sampling was performed during clinical stability, in a well-defined cohort, across three centres applying identical BAL acquisition, processing and storage protocols, and a similar approach to clinical care.

When relative abundance is averaged across the large number of samples in this study (Figure 1), a pattern consistent with that observed in smaller studies is apparent, i.e., the lower airway microbiome is dominated by five major bacterial phyla: Proteobacteria, Firmicutes, Bacteroidetes, Fusobacteria and Actinobacteria (with unassigned phyla a significant sub-group) [4,10,12,13,16,32]. The predominance of Proteobacteria in the lower airway is a common, but not universal, finding, with a wide variation reported in the relative abundance of the other constituent phyla [4,10,12,13,16,32,33]. The lung microbiome in CF and control patients was dominated by well-known CF genera such as *Haemophilus, Staphylococcus, Streptococcus, Pseudomonas*, regularly cultured genera, *Neisseria, Moraxella, Actinomyces, Porphyromonas*, and also newer genera emerging as significant components of the CF microbiome. Many of these ‘emerging genera’ are anaerobes and have been reported previously in CF microbiome studies; *Fusobacterium, Bacteroides, Alistipes, Lachnospiraceae NK4A136 group, Rothia, Prevotella, Gemella, Granulicatella, Leptotrichia, Capnocytophaga, Lachnospiraceae UCG-004, Alloprevotella, Veillonella* [34,35,36,37,38]. While it is now recognised that anaerobes constitute a significant proportion of the CF lung microbiome, the clinical significance of this is still uncertain [39].

The CF and control lung microbiome were broadly similar at the phylum level and in terms of alpha and beta diversity. Unweighted UniFrac analysis revealed only small differences in beta diversity between the cohorts. There are a small number of studies comparing BAL samples between control and CF cohorts, and all are limited by small study numbers and heterogenous cohorts with wide ranging ages and disease severity [6,8,19,33,40]. This makes comparisons with our findings difficult. It has been shown that the lung microbiome in children is similar across different disease cohorts, including CF, yet it is possible to distinguish between the different cohorts based on distinct characteristics of each microbiome [6,8,33,40,41]. Alpha diversity is typically higher in non-CF cohorts [6,8,40] and we did not observe this in our control cohort perhaps due to the young age of the comparison groups. Despite these similarities, we were able to demonstrates that the CF lower airway microbiome in early life has some distinctions from the non-CF. At the genus level, we found nine genera to be of significantly differential abundance between the two cohorts. Six genera were more abundant in control samples, many of which were anaerobes, and two genera, *Staphylococcus* and *Pseudomonas,* were more abundant in CF subjects. Zemanick et al. also found *Staphylococcus* and *Pseudomonas* in greater abundance in the CF lung, and *Prevotella*, *Veillonella*, *Neisseria* and *Porphyromonas* higher in non-CF controls, but, in contrast to our study, *Haemophilus* was similar in both cohorts [8]. Although the CF lower airway microbiome is broadly similar to the controls, we have demonstrated differences between the two microbial communities early in life.

Age was associated with alpha diversity of both the CF and control lung microbiome. However, where increased diversity was associated with age in the control, decreased diversity was associated with age in CF. There were also disparate age-associated phylum-level composition shifts in the CF and control microbiome. In CF, a trend towards increasing abundance of the Proteobacteria phylum (which contains a wide range of Gram-negative pathogens such as *Pseudomonas, Neisseria* and *Haemophilus*) was evident, while the Proteobacteria phylum decreased in abundance in the control microbiome. Similarly, the Firmicutes (*Staphylococcus* and *Streptococcus* containing), Fusobacteria (*Fusobacterium*) and Bacteroidetes (*Porphyromonas, Bacteroides, Prevotella* containing) phyla were disparately associated with age in the CF and control lung microbiome. Distinctions in the CF and control lung microbiome with age were also evident at the genus level. Two genera in the control cohort, *Streptococcus* and *Moraxella*, were less abundance with age. As previously reported by Laguna et al. [13], our study demonstrates that *Streptococcus* is one of the predominant genera that drops in abundance in early childhood in CF. In addition we demonstrate that relative abundance significantly drops for four other genera, *Streptococcus, Veillonella, Neisseria,* and *Porphyromonas*, as the pre-school years advance. In contrast, we found that *Pseudomonas* and *Staphylococcus* were already demonstrating an emerging dominant pattern in this young CF cohort.

To date, there have been limited studies focused on longitudinal changes in the lower airway microbiome in CF. Two studies have reported BAL results from a small number of children at two different time points and report conflicting results [10,12]. Muhlebach et al. demonstrated that five of seven patients maintained the same microbial community type between sampling intervals (ranged from 3.6 to 24 months) [12]. In contrast, Frayman et al. reported that the beta diversity of serial samples from individual subjects was comparable to the beta diversity of samples from different subjects [10]. In our study, sequential annual BAL samples were collected over five years from seven children with CF, aged one to five years. This unique dataset, for the first time, begins to explain the inconsistencies observed in previous studies. Here the lower airway microbiome diversity increased over the first four years of life in young children with CF, before beginning to decline from five years of age. Although this trend is clear for the summary statistics in the group analysis, the changes in phylum-level composition for individuals vary considerably between subjects, and to an equally striking degree, within subjects from year to year. It is clear that sampling at any given time point will result in very different findings. One might also hypothesise that the lower airway microbiome cumulatively establishes itself in the first four years of life, reflected in increasing diversity. Further, this process may begin to reverse or decline by five years of age with the emergence of CF pathogens in the microbiome. Larger cohorts of longitudinal data will be required to corroborate these findings.

Our study demonstrates that unassigned OTUs are prevalent in CF BAL samples. In previous studies, unassigned reads have generally not been reported or they are discarded prior to analysis [42]. We found one exception, where 41% of reads from bronchial brushing samples from school-aged children with CF were reported to correspond to unassigned OTUs [32]. In the present study, comparison of CF versus control BAL samples reveals that 15–22% (higher in the CF cohort) of the relative abundance of the lower airway constitutes unassigned OTUs. These unassigned reads are unlikely to be from human DNA sources as we performed 16S rDNA sequencing. They are equally unlikely to be the result of non-specific amplification as the quality control would rule out any poor quality reads. This suggests that, as yet, undefined microbes are present in the lung.

Airway inflammation is a central component in the pathogenesis of CF lung disease. If the microbiome plays a role in CF lung disease, one would expect it to influence, or be influenced by, airway inflammation. Although we found that in children with lower diversity, airway inflammation, as measured by NE and IL-8, was higher, our study was similar to others in that the association was not consistent across different inflammatory markers and different measures of diversity [10,11,12,13]. Further studies are required to understand if the association becomes more pronounced over time and, if so, to attempt to determine causality.

Surprisingly, antibiotic exposure did not have a detectable effect on the lower airway microbiome. Marsh et al. also failed to demonstrate an effect of current antibiotic use on the lung microbiome of young, non-CF, children [41]. While Zemanick et al. did show a clear correlation with antibiotic use and reduced diversity, a large proportion of the cohort from that study were on antibiotics as the BALs were performed for clinical reasons in symptomatic subjects, which included a significant number of adult CF patients [8]. Pittman et al. demonstrated lower diversity in infants on anti-Staphylococcal antibiotics, a finding we did not replicate, but which may be partly explained by the use of a broad spectrum antibiotic (amoxicillin-clavunate) in the Pittman study compared with flucloxacillin in our study [11]. A relatively small number of infants in our cohort had quite extensive exposure to intravenous and/or oral antibiotics but this did not differentiate their microbiome from those with little or no antibiotic exposure. It may be that, as children were required to be at a stable baseline for bronchoscopy, previous perturbations of the microbiome had resolved at the time of sample acquisition [5].

This study has several limitations. Not all subjects had samples taken from one year of age. This reflected different clinical practices across the different centres. Some subjects did not have a BAL performed every year. As the SHIELD CF study is based on a clinical BAL surveillance programme, patients could have their procedure deferred or cancelled if the attending physician did not feel they were well at the time. Recording prior antibiotic exposure proved difficult to capture as it is often incompletely recorded in clinical notes. We cross-checked chart records with available pharmacy and prescription records but the information is likely incomplete for some patients. The control cohort consisted of children having a BAL for clinical reasons, and therefore, their underlying condition may have influenced the findings. It was not possible to obtain sequence data that passed our stringent quality controls for all samples. However, at 87%, our sequencing success rate is considerably higher than previous publications [8,10,11,32]. Despite these limitations, our study has significant strengths. It is the largest study of the CF lower airway microbiome of young children performed to date, that has both a large control cohort for comparison and longitudinal samples. We included negative controls of the sampling process, DNA extraction, library preparation and sequencing protocols in line with the more robust lung microbiome studies [8,10]. There was a pattern of agreement between our culture data and sequencing data, keeping in mind that sequencing is a more sensitive method of detection. Most other CF microbiome studies use sputum samples, arguably a sample with upper airway representation, and many using BAL have not used the same bronchoscopy methods used here.

In summary, we have demonstrated that the CF lower airway microbiome in infants and pre-school-aged children with CF is different to the lower airway of non-CF children. The CF lower airway microbiome becomes less diverse with age, in contrast to the control lower airway microbiome. A lower relative abundance of the *Streptococcus* genus and several anaerobes and increasing relative abundance of the *Pseudomonas* and *Staphylococcus* genera are associated with age in CF. There are significant differences in microbiome composition between individuals and within individuals over time. The lower airway microbiome in clinically stable children with CF was not influenced by prior antibiotic use in this study, but early changes in its diversity are associated with subtle evidence of airway inflammation.

## Figures and Tables

**Table 2:** Correlations between inflammation and measures of alpha diversity.

Marker	Statistic	chao1	Simpson	Shannon	PD Whole Tree	Observed Species
log10(ANC)	Adjusted *p*-value	0.820	0.067	0.261	0.927	0.844
	*R*-value	0.044	−0.164	−0.102	−0.013	0.038
log10(IL-8)	Adjusted *p*-value	0.927	***0.008***	***0.037***	0.067	0.927
	*R*-value	−0.011	−0.211	−0.160	0.139	−0.006
NE	Adjusted *p*-value	0.224	***0.037***	***0.037***	0.400	0.224
	*R*-value	-0.099	−0.171	−0.159	−0.070	−0.097
Total cell count	Adjusted *p*-value	0.927	0.215	0.261	0.927	0.927
	*R*-value	0.013	−0.117	−0.097	0.014	0.006

Definitions of abbreviations: log10 = log to the base 10, ANC = absolute neutrophil count, IL-8 = interleukin 8, and NE = neutrophil elastase. Significance at <0.05 is indicated in italics.

## Data Availability

Data sets have been deposited in the European Nucleotide Archive under project number PRJEB28588.

## Supplementary Materials

The following are available online at https://www.mdpi.com/2076-2607/9/3/492/s1; Figure S1: Sample processing flow diagram. Figure S2: Correspondence between culture and sequencing data. The mean relative abundance of important CF genera as detected by 16S rRNA gene sequencing is presented across culture positive and culture negative samples. Figure S3. Line graphs of relative abundance for each of the phyla included in Figure 3, at ages 1 to 6 years old. Error bars show standard error. Table S1. Linear discriminant analysis (LDA) effect size (LEfSe) was used to identify differentially abundant taxa; an alpha value of ≤0.05 and LEfSe of ≥4.0 was used as the threshold for significance. Table S2. Correlations between alpha diversity and cumulative days on antibiotics in the preceding year (oral and/or intravenous) prior to BAL. Table S3. Numbers of samples per age group. Supplementary File F1: Total number of sequence reads and assigned taxa in negative controls. Supplementary File F2: Decontam outcomes from negative controls.

## Appendix A. Supplementary Methods

DNA extraction. The DNA extraction process was performed as previously described [19]. Briefly, frozen BAL samples were thawed on ice and centrifuged for 10 min at 10,000× *g*. The pelleted material was resuspended in 600 µL of buffer RLT (Qiagen DNA/ RNA AllPrep Mini kit) and bead beating performed (0.3 g of 180 µM acid washed glass beads; Sigma Aldrich, Germany) for 30 sec at 5.5 m·sec^−1^. Supernatant was transferred to a Qiagen DNA spin column and DNA extracted using the Qiagen DNA/ RNA AllPrep Mini kit. DNA quantity was determined using the Quant-iT PicoGreen dsDNA assay kit (Invitrogen, Renfrew, UK) following the manufacturer’s instructions. A saline control sample was included where the entire DNA extraction process was performed with 1 mL of 0.9% NaCl as the start material. The DNA extraction protocol was also performed on a subset of bronchoscope controls; 5 mL of sterile 0.9% NaCl suctioned through the bronchoscopes and captured in a BAL sample pot prior to the bronchoscopic procedure commencing.

16S rRNA gene sequencing. DNA was normalised and 16S metagenomic libraries were prepared using primers to amplify the V3–V4 region of the 16S gene [20], with Illumina adaptors incorporate as described in the Illumina 16S Metagenomic Library Preparation guide with the following exceptions; Kapa Robust (Kapa Biosystems/ Merck, Watford, UK) was used in place of Kapa Hifi, the first PCR reaction was performed in a total volume of 50 μL instead of 25 μL and the number of cycles used in the first PCR was increased from 25 cycles to 30. The volume of AMPure XP beads (New England Biolab (NEB), Hitchin, UK) used in the initial clean-up was scaled up accordingly. Following index PCR and purification, the products were quantified using the Qubit high-sensitivity DNA kit (Thermo Fisher Scientific, Bleiswijk, Netherlands) and pooled equimolarly with approximately 50 samples per pool. The pooled libraries were assessed by an Agilent high-sensitivity DNA kit and quantified by qPCR using the Kapa Quantification kit for Illumina (Kapa Biosystems/ Merck millipore, Watford, UK) according to the manufacturer’s guidelines. Libraries were then diluted and denatured following Illumina guidelines and sequenced on the Illumina MiSeq using the V2 500 cycle kit.

Control samples. A saline control sample was included where the entire DNA extraction process was performed with 1 mL of 0.9% NaCl as the start material. The DNA extraction protocol was also performed on a subset of bronchoscope controls; 5 mL of sterile 0.9% NaCl suctioned through the bronchoscopes and captured in a BAL sample pot prior to the bronchoscopic procedure commencing. These samples were carried through the entire protocol from DNA extraction to sequencing.

Avoidance of upper airway contamination. Because PCR based techniques have such high-sensitivity contamination of samples from the upper airway can distort attempts to accurately define the lower airway. By employing an identical protocol in all centres, which involved passing the bronchoscope through a LMA and avoiding suctioning until the scope had passed the carina, we believe we obtained samples truly representative of lung microbiome.

## References

[1] Elborn J.S. (2016). Cystic fibrosis. Lancet.

[2] Ranganathan S.C., Hall G.L., Sly P.D., Stick S.M., Douglas T.A. (2017). Early Lung Disease in Infants and Preschool Children with Cystic Fibrosis. What Have We Learned and What Should We Do about It?. Am. J. Respir. Crit. Care Med..

[3] Lipuma J.J. (2010). The changing microbial epidemiology in cystic fibrosis. Clin. Microbiol. Rev..

[4] Cox M.J., Allgaier M., Taylor B., Baek M.S., Huang Y.J., Daly R.A., Karaoz U., Andersen G.L., Brown R., Fujimura K.E. (2010). Airway microbiota and pathogen abundance in age-stratified cystic fibrosis patients. PLoS ONE.

[5] Fodor A.A., Klem E.R., Gilpin D.F., Elborn J.S., Boucher R.C., Tunney M.M., Wolfgang M.C. (2012). The adult cystic fibrosis airway microbiota is stable over time and infection type, and highly resilient to antibiotic treatment of exacerbations. PLoS ONE.

[6] Harris J.K., De Groote M.A., Sagel S.D., Zemanick E.T., Kapsner R., Penvari C., Kaess H., Deterding R.R., Accurso F.J., Pace N.R. (2007). Molecular identification of bacteria in bronchoalveolar lavage fluid from children with cystic fibrosis. Proc. Natl. Acad. Sci. USA.

[7] Stressmann F.A., Rogers G.B., van der Gast C.J., Marsh P., Vermeer L.S., Carroll M.P., Hoffman L., Daniels T.W., Patel N., Forbes B. (2012). Long-term cultivation-independent microbial diversity analysis demonstrates that bacterial communities infecting the adult cystic fibrosis lung show stability and resilience. Thorax.

[8] Zemanick E.T., Wagner B.D., Robertson C.E., Ahrens R.C., Chmiel J.F., Clancy J.P., Gibson R.L., Harris W.T., Kurland G., Laguna T.A. (2017). Airway microbiota across age and disease spectrum in cystic fibrosis. Eur. Respir. J..

[9] Rabin H.R., Surette M.G. (2012). The cystic fibrosis airway microbiome. Curr. Opin. Pulm. Med..

[10] Frayman K.B., Armstrong D.S., Carzino R., Ferkol T.W., Grimwood K., Storch G.A., Teo S.M., Wylie K.M., Ranganathan S.C. (2017). The lower airway microbiota in early cystic fibrosis lung disease: A longitudinal analysis. Thorax.

[11] Pittman J.E., Wylie K.M., Akers K., Storch G.A., Hatch J., Quante J., Frayman K.B., Clarke N., Davis M., Stick S.M. (2017). Association of Antibiotics, Airway Microbiome, and Inflammation in Infants with Cystic Fibrosis. Ann. Am. Thorac. Soc..

[12] Muhlebach M.S., Zorn B.T., Esther C.R., Hatch J.E., Murray C.P., Turkovic L., Ranganathan S.C., Boucher R.C., Stick S.M., Wolfgang M.C. (2018). Initial acquisition and succession of the cystic fibrosis lung microbiome is associated with disease progression in infants and preschool children. PLoS Pathog..

[13] Laguna T.A., Wagner B.D., Williams C.B., Stevens M.J., Robertson C.E., Welchlin C.W., Moen C.E., Zemanick E.T., Harris J.K. (2016). Airway Microbiota in Bronchoalveolar Lavage Fluid from Clinically Well Infants with Cystic Fibrosis. PLoS ONE.

[14] Carmody L.A., Zhao J., Schloss P.D., Petrosino J.F., Murray S., Young V.B., Li J.Z., LiPuma J.J. (2013). Changes in cystic fibrosis airway microbiota at pulmonary exacerbation. Ann. Am. Thorac. Soc..

[15] Zhao J., Schloss P.D., Kalikin L.M., Carmody L.A., Foster B.K., Petrosino J.F., Cavalcoli J.D., VanDevanter D.R., Murray S., Li J.Z. (2012). Decade-long bacterial community dynamics in cystic fibrosis airways. Proc. Natl. Acad. Sci. USA.

[16] Goddard A.F., Staudinger B.J., Dowd S.E., Joshi-Datar A., Wolcott R.D., Aitken M.L., Fligner C.L., Singh P.K. (2012). Direct sampling of cystic fibrosis lungs indicates that DNA-based analyses of upper-airway specimens can misrepresent lung microbiota. Proc. Natl. Acad. Sci. USA.

[17] Bacci G., Mengoni A., Fiscarelli E., Segata N., Taccetti G., Dolce D., Paganin P., Morelli P., Tuccio V., De Alessandri A. (2017). A Different Microbiome Gene Repertoire in the Airways of Cystic Fibrosis Patients with Severe Lung Disease. Int. J. Mol. Sci..

[18] Editors P.M. (2014). Observational studies: Getting clear about transparency. PLoS Med..

[19] Renwick J., McNally P., John B., DeSantis T., Linnane B., Murphy P., Shield C.F. (2014). The microbial community of the cystic fibrosis airway is disrupted in early life. PLoS ONE.

[20] Klindworth A., Pruesse E., Schweer T., Peplies J., Quast C., Horn M., Glockner F.O. (2013). Evaluation of general 16S ribosomal RNA gene PCR primers for classical and next-generation sequencing-based diversity studies. Nucleic Acids Res..

[21] Walsh A.M., Crispie F., Daari K., O’Sullivan O., Martin J.C., Arthur C.T., Claesson M.J., Scott K.P., Cotter P.D. (2017). Strain-Level Metagenomic Analysis of the Fermented Dairy Beverage Nunu Highlights Potential Food Safety Risks. Appl. Environ. Microbiol..

[22] Caporaso J.G., Bittinger K., Bushman F.D., DeSantis T.Z., Andersen G.L., Knight R. (2010). PyNAST: A flexible tool for aligning sequences to a template alignment. Bioinformatics.

[23] Altschul S.F., Gish W., Miller W., Myers E.W., Lipman D.J. (1990). Basic local alignment search tool. J. Mol. Biol..

[24] Quast C., Pruesse E., Yilmaz P., Gerken J., Schweer T., Yarza P., Peplies J., Glockner F.O. (2013). The SILVA ribosomal RNA gene database project: Improved data processing and web-based tools. Nucleic Acids Res..

[25] Wang Q., Garrity G.M., Tiedje J.M., Cole J.R. (2007). Naive Bayesian classifier for rapid assignment of rRNA sequences into the new bacterial taxonomy. Appl. Environ. Microbiol..

[26] DeSantis T.Z., Hugenholtz P., Larsen N., Rojas M., Brodie E.L., Keller K., Huber T., Dalevi D., Hu P., Andersen G.L. (2006). Greengenes, a chimera-checked 16S rRNA gene database and workbench compatible with ARB. Appl. Environ. Microbiol..

[27] McMurdie P.J., Holmes S. (2013). phyloseq: An R package for reproducible interactive analysis and graphics of microbiome census data. PLoS ONE.

[28] Oksanen J. (2007). The vegan package. Community Ecol. Package.

[29] Harrell F.E., Harrell M.F.E. (2017). Package ‘Hmisc’.

[30] Segata N., Izard J., Waldron L., Gevers D., Miropolsky L., Garrett W.S., Huttenhower C. (2011). Metagenomic biomarker discovery and explanation. Genome Biol..

[31] Wickham H. (2016). ggplot2: Elegant Graphics for Data Analysis.

[32] An S.Q., Warris A., Turner S. (2018). Microbiome characteristics of induced sputum compared to bronchial fluid and upper airway samples. Pediatr. Pulmonol..

[33] Kloepfer K.M., Deschamp A.R., Ross S.E., Peterson-Carmichael S.L., Hemmerich C.M., Rusch D.B., Davis S.D. (2018). In children, the microbiota of the nasopharynx and bronchoalveolar lavage fluid are both similar and different. Pediatr. Pulmonol..

[34] Acosta N., Whelan F.J., Somayaji R., Poonja A., Surette M.G., Rabin H.R., Parkins M.D. (2017). The Evolving Cystic Fibrosis Microbiome: A Comparative Cohort Study Spanning 16 Years. Ann. Am. Thorac. Soc..

[35] Rogers G.B., Hart C.A., Mason J.R., Hughes M., Walshaw M.J., Bruce K.D. (2003). Bacterial diversity in cases of lung infection in cystic fibrosis patients: 16S ribosomal DNA (rDNA) length heterogeneity PCR and 16S rDNA terminal restriction fragment length polymorphism profiling. J. Clin. Microbiol..

[36] Coburn B., Wang P.W., Diaz Caballero J., Clark S.T., Brahma V., Donaldson S., Zhang Y., Surendra A., Gong Y., Elizabeth Tullis D. (2015). Lung microbiota across age and disease stage in cystic fibrosis. Sci. Rep..

[37] Surette M.G. (2014). The cystic fibrosis lung microbiome. Ann. Am. Thorac. Soc..

[38] De Dios Caballero J., Vida R., Cobo M., Maiz L., Suarez L., Galeano J., Baquero F., Canton R., Del Campo R. (2017). Individual Patterns of Complexity in Cystic Fibrosis Lung Microbiota, Including Predator Bacteria, over a 1-Year Period. MBio.

[39] Sherrard L.J., Bell S.C., Tunney M.M. (2016). The role of anaerobic bacteria in the cystic fibrosis airway. Curr. Opin. Pulm. Med..

[40] Van der Gast C.J., Cuthbertson L., Rogers G.B., Pope C., Marsh R.L., Redding G.J., Bruce K.D., Chang A.B., Hoffman L.R. (2014). Three clinically distinct chronic pediatric airway infections share a common core microbiota. Ann. Am. Thorac. Soc..

[41] Marsh R.L., Kaestli M., Chang A.B., Binks M.J., Pope C.E., Hoffman L.R., Smith-Vaughan H.C. (2016). The microbiota in bronchoalveolar lavage from young children with chronic lung disease includes taxa present in both the oropharynx and nasopharynx. Microbiome.

[42] Jervis-Bardy J., Leong L.E., Marri S., Smith R.J., Choo J.M., Smith-Vaughan H.C., Nosworthy E., Morris P.S., O’Leary S., Rogers G.B. (2015). Deriving accurate microbiota profiles from human samples with low bacterial content through post-sequencing processing of Illumina MiSeq data. Microbiome.

