# Supplementary material for: The Lung Microbiome in Young Children with Cystic Fibrosis: A Prospective Cohort Study
Source: Microorganisms. 2021 Feb 26;9(3):492. doi: 10.3390/microorganisms9030492 (PMC7996874; doi:10.3390/microorganisms9030492)
Supplement: Supplementary file 1 [file microorganisms-09-00492-s001.zip › microorganisms-1099512-s/Supplementary data/Table S2.pdf]

Table S2. Correlations between alpha diversity and cumulative days on antibiotics in the preceding year (oral and/or intravenous) prior to BAL.

**Index**	**Statistic**	**Cumulative days on antibiotics**
Chao1	R-value	-0.027
	Unadjusted P-value	0.777
Observed species	R-value	-0.031
	Unadjusted P-value	0.743
PD whole tree	R-value	-0.151
	Unadjusted P-value	0.109
Shannon	R-value	0.028
	Unadjusted P-value	0.770
Simpson	R-value	0.016
	Unadjusted P-value	0.862

Excluding long-term prophylactic antibiotics (flucloxacillin or azithromycin)
