# Supplementary material for: The Lung Microbiome in Young Children with Cystic Fibrosis: A Prospective Cohort Study
Source: Microorganisms. 2021 Feb 26;9(3):492. doi: 10.3390/microorganisms9030492 (PMC7996874; doi:10.3390/microorganisms9030492)
Supplement: Supplementary file 1 [file microorganisms-09-00492-s001.zip › microorganisms-1099512-s/Supplementary data/Table S3.pdf]

Table S3. Numbers of samples per age group

	Number of samples per age group	
	CF	N
<1 years	1	0
1 years	42	7
2 years	47	4
3 years	56	5
4 years	50	7
5 years	44	3
6 years	26	4
>6 years	7	21
