# Supplementary figures and images for: The Lung Microbiome in Young Children with Cystic Fibrosis: A Prospective Cohort Study
Source: Microorganisms. 2021 Feb 26;9(3):492. doi: 10.3390/microorganisms9030492 (PMC7996874; doi:10.3390/microorganisms9030492)

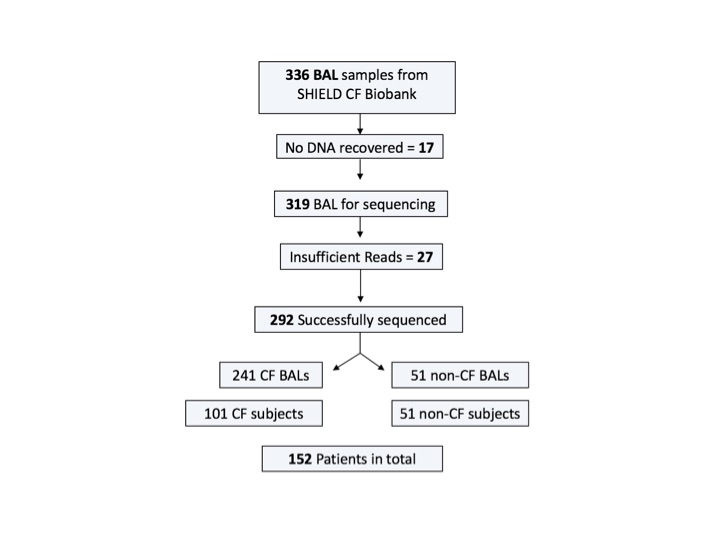

Supplement: Supplementary file 1 [file microorganisms-09-00492-s001.zip › microorganisms-1099512-s/Supplementary data/Figure S1.jpg]

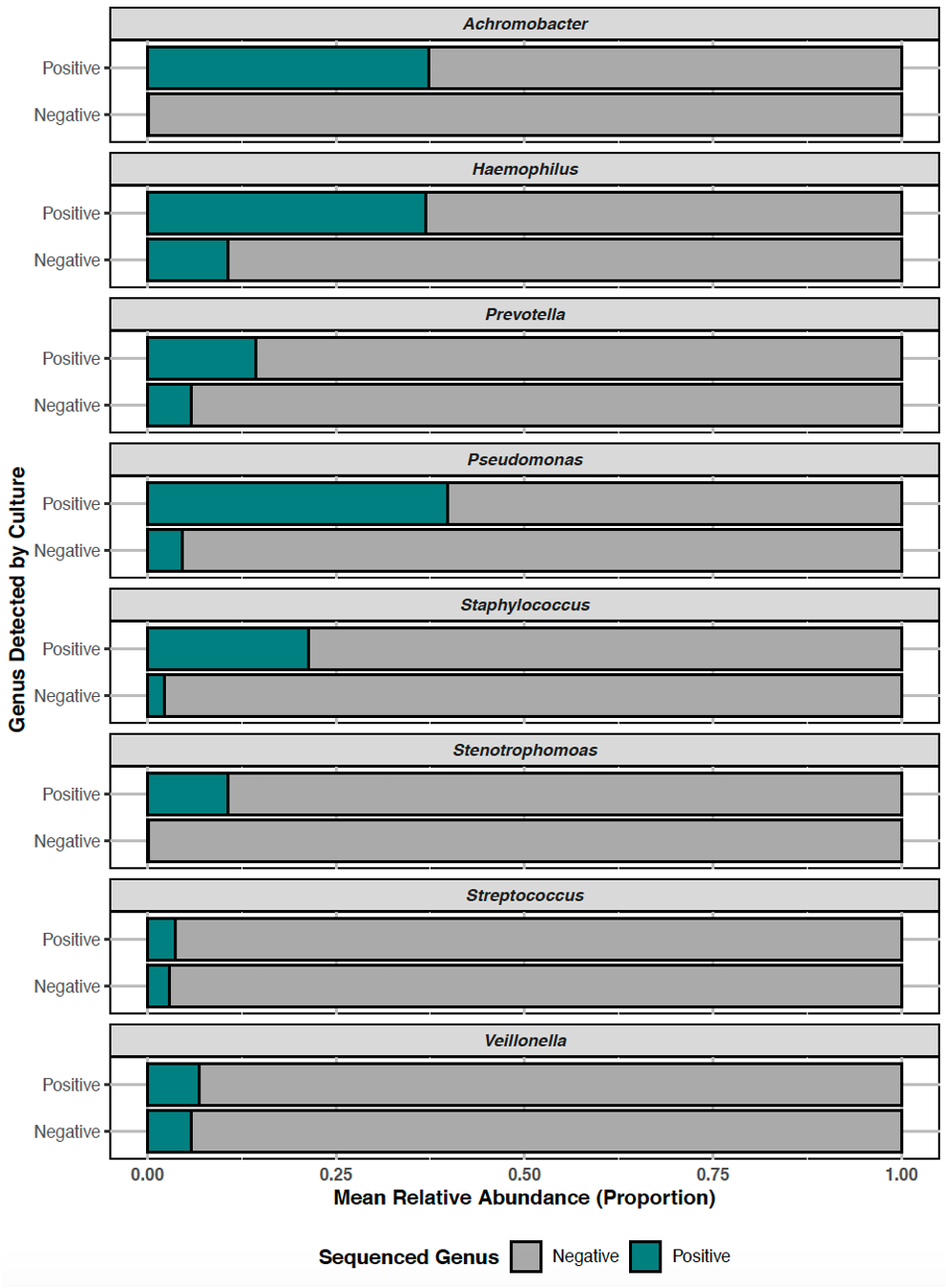

Supplement: Supplementary file 1 [file microorganisms-09-00492-s001.zip › microorganisms-1099512-s/Supplementary data/Figure S2.png]

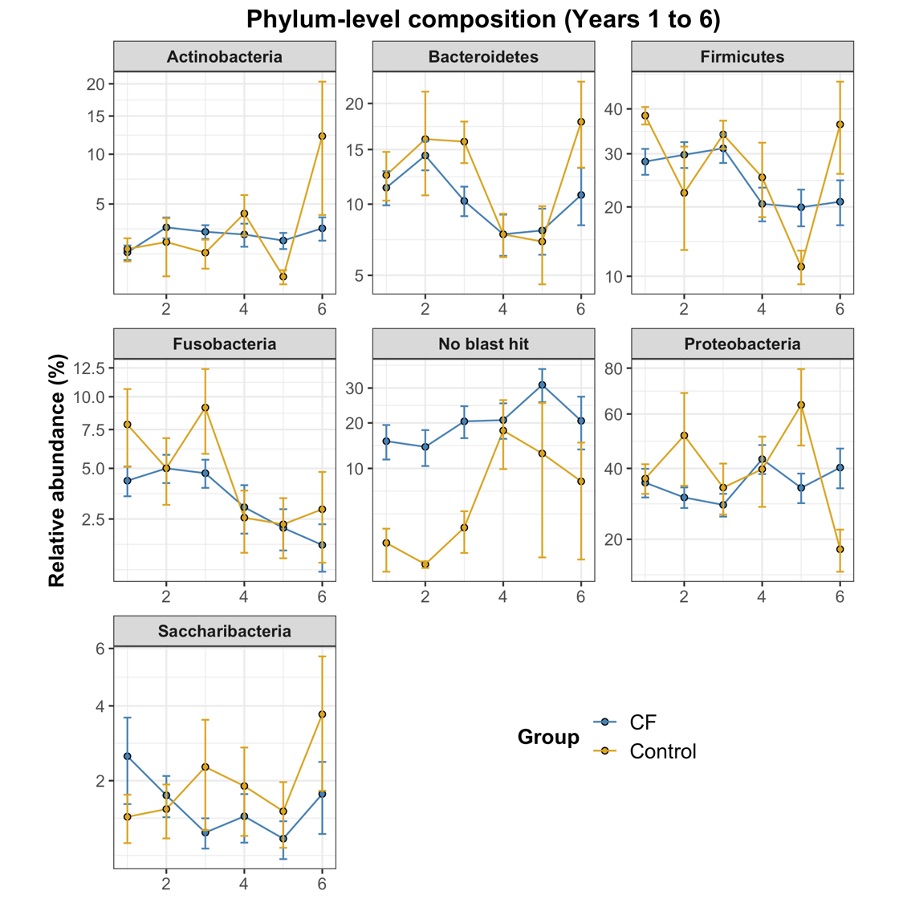

Supplement: Supplementary file 1 [file microorganisms-09-00492-s001.zip › microorganisms-1099512-s/Supplementary data/Figure S3 .jpg]

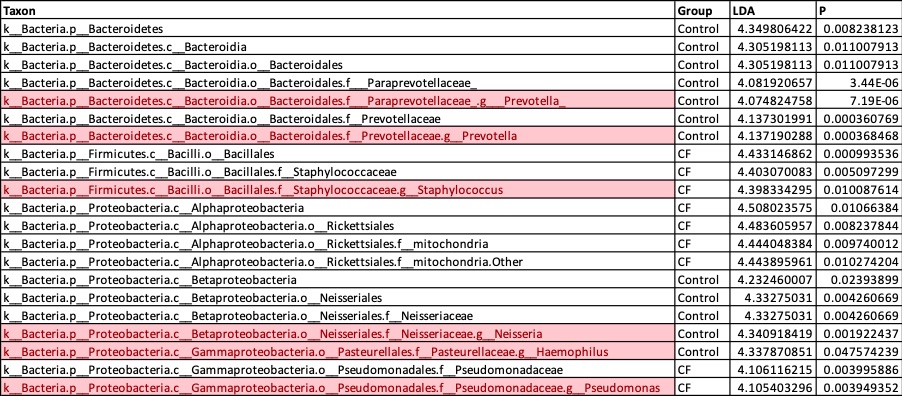

Supplement: Supplementary file 1 [file microorganisms-09-00492-s001.zip › microorganisms-1099512-s/Supplementary data/Table S1.jpg]
